# Factors Associated with Hospital Length of Stay and Intensive Care Utilization Among Pediatric COVID-19 Patients in Southern Nevada: A Multivariate Analysis

**DOI:** 10.3390/children12030332

**Published:** 2025-03-06

**Authors:** Erika Marquez, Amanda Haboush-Deloye, Jihye Kim, Erick B López, Anil T. Mangla, Binita Adhikari, Jay J. Shen

**Affiliations:** 1 School of Public Health, University of Nevada Las Vegas, Las Vegas, NV 89154, USA; 2 Office of Disease Surveillance and Control, Southern Nevada Health District, Las Vegas, NV 89107, USA

**Keywords:** children, COVID-19, social vulnerability index (SVI)

## Abstract

The COVID-19 pandemic has revealed significant disparities in health outcomes across various populations, with children being no exception. **Objective:** This study aimed to identify factors associated with hospital length of stay and intensive care unit (ICU) utilization among children hospitalized with COVID-19. **Methods:** The project evaluated inpatient COVID-19 hospitalization data of children aged 0 to 17 years between 2020 and 2021 with a positive PCR COVID-19 test 14 days prior to or during hospitalization. Using a multivariate linear regression model, hospital length of stay and ICU utilization were evaluated by sociodemographic factors, including age, gender, race/ethnicity, primary payer status, comorbidities, CDC Social Vulnerability Index (SVI), and clinical factors. **Results:** Among 376 hospitalized pediatric patients, 62.2% were non-White minorities, 4.3% had at least one comorbidity, and 58.5% were covered by public insurance. Additionally, 67.6% scored high on the SVI. The average hospital stay was 3.89 days (standard deviation (SD) = 4.8), and 25% of children utilized the ICU during their hospitalization (SD = 0.43). After adjusting for sociodemographic and clinical characteristics, minority patients were more likely to have a longer length of stay by 1.09 days compared to White patients. Minority patients were also 72% more likely to use the ICU than White patients. **Conclusions:** These findings demonstrate that non-White children experience more severe outcomes related to COVID-19, supporting the need for culturally specific mitigation and intervention strategies for children and families during a pandemic.

## 1. Introduction

The COVID-19 pandemic, caused by the SARS-CoV-2 coronavirus, has triggered widespread disruption across the globe and widespread health effects. According to the Centers for Disease Control and Prevention (CDC), in the United States, approximately 7 million people have been hospitalized, and approximately 1.2 million deaths have been attributed to COVID-19 as of March 2024 [1]. The pandemic had both direct and indirect impacts on family dynamics due to job losses and the loss of loved ones. These challenges significantly affected mental health, disrupted children’s education, and impacted the overall well-being of families and children [2]. Although the impacts have been felt across all communities, families from lower-income and racial and ethnic minority backgrounds have disproportionately experienced illness and have faced greater difficulties dealing with the social impacts of the pandemic compared to other groups [2,3].

COVID-19 mortality and morbidity in adults are well documented, but less is known about the risks of severe disease among children [4,5]. While children had a significantly lower prevalence of COVID-19 infection and were less likely to develop severe disease, children were hospitalized with or developed conditions such as multisystem inflammatory syndrome [6,7,8], and approximately 2000 children under the age of 17 have had a COVID-19-related death from January 2020 to June 2023 [9]. Emerging data have shown that while children typically experience milder illness compared to adults, some pediatric populations are still at risk of severe outcomes requiring hospitalization. For example, a scoping review of studies suggested that higher rates of severe illness were found among children with certain underlying medical conditions like obesity, diabetes, and immunosuppression [10]. In addition to medical risk factors, social determinants of health (SDoHs) played a significant role in COVID-19 severity and outcomes among children [11].

SDoHs influence how well individuals and communities can respond to and recover from health threats, often making some populations more susceptible to adverse health outcomes. Social vulnerability refers to the circumstances shaped by physical, social, economic, and environmental factors or processes that further heighten this vulnerability [12]. Communities in socially vulnerable areas are especially at risk during public health emergencies like COVID-19. County-level research showed that these communities had a greater likelihood of COVID-19 infection and higher mortality rates [13,14]. Notably, socially vulnerable children exhibited poorer health outcomes when affected by COVID-19 [11]. Thus, identifying such a population during a time of crisis is critical. The CDC’s Social Vulnerability Index (CDC-SVI) has been used to help public health officials and emergency planners find vulnerable communities to plan emergency response and communication [15]. Several studies demonstrated that high CDC-SVI is linked to poorer health outcomes in children. For example, prior research has shown the association between a higher CDC-SVI and greater risks of asthma, obesity, and cardiovascular disease [16,17,18]. Moreover, children residing in high CDC-SVI areas were more likely to suffer from postoperative complications [19]. Effective public health prevention and mitigation efforts can be devised to safeguard this vulnerable population during pandemics and increase our understanding of how social determinants impact COVID-19 outcomes in children.

This project aimed to identify factors, including social vulnerability, associated with outcomes among children hospitalized with COVID-19. Severity was gauged through indicators such as length of hospital stay and intensive care unit (ICU) admission rates. Understanding factors associated with severe child outcomes is essential, especially in vulnerable populations, to prepare for future pandemics.

## 2. Materials and Methods

### 2.1. Data

This was a community public health practice and improvement project. We used patient records from 2020 to 2021 from the following two data sources: (1) Nevada hospital discharge dataset from the Center for Health Information Analysis (CHIA) and (2) Southern Nevada Health District (SNHD)’s EpiTrax disease surveillance system data. Nevada hospital discharge data included inpatient hospital discharge data derived from health facility billing records, which contained patient-level billing data, demographics (e.g., age, gender, race/ethnicity), primary payer, diagnostic and procedural details, length of stay, and a set of binary indicators for comorbidities defined according to the modified Elixhauser algorithm. The EpiTrax data were used to determine COVID-19 testing status and mortality outcomes.

We also used the CDC-SVI data developed by the CDC, which is a quantitative index to identify communities that may need additional support and resources before, during, and after natural disasters or emergency events [15,20]. Overall vulnerability was measured based on the neighborhood’s socioeconomic characteristics, household composition and disability, minority status and language, and housing and transportation. We linked the SVI data to patients’ records using residential ZIP Codes. Data cleaning and processing were conducted using SAS v9.4, and data analysis was conducted using Stata 18.

### 2.2. Study Sample

Children were included in the project if they met the following criteria: (a) aged 0 to 17 years, (b) were Clark County residents, and (c) had an inpatient COVID-19 hospitalization in Nevada between 2020 and 2021 and a positive PCR COVID-19 test 14 days before or during hospitalization. Hospitalizations with COVID-19-related diagnoses were defined based on relevant ICD-10 codes consistent with the CDC’s definition of COVID-19 hospitalization [9].

### 2.3. Measures

#### 2.3.1. Dependent Variables

There were two outcome variables: (1) the number of days the child was hospitalized (length of stay) and (2) a binary indicator for ICU utilization.

#### 2.3.2. Independent Variables

The independent variables included in the model were selected based on clinical relevance, prior literature, statistical considerations, and data availability. Demographic factors were included in the analyses to test the association with severe COVID-19-related outcomes: age group, sex, and race/ethnicity. As a proxy of the family’s income level, the primary payer type was included. Indicators for clinical factors included having at least one comorbidity, having COVID-19 and other related diagnoses as a primary diagnosis, and having COVID-19 symptoms (i.e., cough, fever, shortness of breath) or any associated conditions (e.g., unspecified acute lower respiratory infection, other specified respiratory disorders, pneumonia due to coronavirus disease 2019). Comorbidities were relatively rare in our sample. Therefore, we created an indicator for having at least one comorbidity. For each ZIP Code Tabulation Area (ZCTA), the SVI was categorized into two groups based on the overall social vulnerability percentile rank: low to moderate vulnerability (for percentile ranks less than or equal to 0.666) and high vulnerability (for percentile ranks greater than 0.666).

### 2.4. Statistical Analyses

All models included patients’ sociodemographic and clinical characteristics. Sociodemographics included age group (0 to 4 years; 5 to 12 years; and 13 to 17 years); sex (female and male); race/ethnicity (non-Hispanic White and racial and ethnic minority groups were combined, including Asian, Black, Native American, Hispanic, and other races); primary payer type (Medicaid, Civilian Health and Medical Program of the Department of Veterans Affairs (CHAMPVA), or Civilian Health and Medical Program of the Uniformed Services (CHAMPUS); commercial insurer, PPO, or HMO; and other (charity, self-pay, or miscellaneous)); and ZIP Code-level SVI (low to moderate vulnerability and high vulnerability). Clinical characteristics included an indicator for having at least one comorbidity, having COVID-19 and other related diagnoses as a primary diagnosis, and having COVID-19 symptoms or any associated conditions (e.g., unspecified acute lower respiratory infection, other specified respiratory disorders, pneumonia due to COVID-19). COVID-19 symptoms were included as an independent variable because not all patients with COVID-19 with a primary diagnosis exhibited the same clinical manifestations.

First, the study population characteristics were descriptively presented. Then, a multivariate linear regression model estimated the association between the length of stay and predictor variables. Although the normality test of the length of stay indicated some degree of skewness, we did not transform data for a more intuitive clinical interpretation of the results and robustness due to the central limit theorem given our sample size. The association between the ICU utilization and predictor variables was estimated based on a multivariate logistic regression model, where results were presented with an adjusted odds ratio (aOR). For both models, standard errors were clustered at the inpatient facility level. Year-quarter fixed effects were included to control for unobserved quarter-specific characteristics (i.e., seasonality). Multicollinearity among the independent variables was tested using variance inflation factor (VIF) analysis. The VIF values for age, gender, race, payer, comorbidity, and SVI, as well as for COVID-19 as a primary diagnosis and COVID-19 symptoms, were all below 5, indicating no severe multicollinearity. Observations with missing values (17.4%) were excluded from the analyses.

## 3. Results

There were 376 observations with populated values for the length of stay variable. The mean number of days in the hospital was 3.89 days, with a standard deviation of 4.81. There were 235 observations with non-missing ICU utilization, where 58 children utilized the ICU during their hospitalization (25%). 

Table 1 represents the descriptive characteristics of patients. Among children with at least one inpatient COVID-19 admission in our sample, 40.69%, 27.93%, and 31.38% were aged 0–4, 5–12, and 13–17 years, respectively. In our sample, 62.23% of the children were racial and ethnic minorities, 28.72% had COVID-19-related primary diagnosis, 34.84% had COVID-19 symptoms and related conditions, and 67.55% resided in a ZIP Code with a high SVI. A total of 58.51% of children were enrolled in either Medicaid, CHAMPUS, or CHAMPVA, and 36.44% were enrolled in either a commercial insurer, PPO, or HMO.

Table 2 documents the results of the multivariate regression models after adjusting for the aforementioned sociodemographic and clinical characteristics. Minority patients were associated with a longer length of stay by 1.09 days (95% confidence interval (CI): 0.42, 1.76) compared to non-Hispanic White patients. Additionally, having COVID-19 symptoms or any related conditions was associated with a longer length of stay of about 3.55 days (95% CI: 0.19, 6.91). The ZIP Code-level SVI was not significantly associated with length of stay.

The multivariate logistic regression model showed that minority patients were associated with 72 percent (95% CI: 1.09, 2.71) higher odds of using the ICU compared to non-Hispanic White patients. Although the association did not reach conventional statistical significance (*p* = 0.054), children enrolled in Medicaid/CHAMPUS/CHAMPVA were marginally associated with 2.44 times (95% CI: 0.99, 6.03) higher odds of using the ICU compared to those with a commercial insurer/PPO/HMO. We found no significant association between the ZIP Code-level SVI and ICU usage.

## 4. Discussion

COVID-19 has had a profound and widespread impact on population health across the globe. While our understanding of the impacts on adults is relatively clear, less is known about the factors associated with outcomes among children hospitalized due to COVID-19. This project aimed to expand our understanding by examining the factors associated with the length of stay and ICU utilization among children hospitalized with COVID-19 in Clark County, Nevada.

This project found that minority patients had a longer length of stay by 1.09 days and were 72% more likely to use intensive care compared to patients who were non-Hispanic White. Other studies have also found that there is an increase in the severity of COVID-19 for hospitalized pediatric patients who were non-White [21,22,23]. For example, Bandi, Nevid, and Mahdavnia (2020) found that 80% of children hospitalized and all ICU-admitted patients were African American [21]. Another study with a sample of U.S. children with COVID-19 found that age, race/ethnicity, and medical conditions were significantly associated with hospitalization. Those who experienced the highest COVID-19-related mortality were African American children and those with a comorbidity [22]. Similar findings were reported in a study based in Brazil, where there was an increased likelihood of death for those of Indigenous ethnicity compared to those who were White [5].

Our project identified that children on Medicaid or other government-type insurance were marginally associated with ICU usage than those with commercial insurance. However, analyses of insurance type and the impact on hospitalization severity are relatively rare in pediatric studies, and a study found that across 45 U.S. children’s hospitals between April 2020 and September 2020, enrollment in private insurance increased the odds of hospitalization compared to those who were discharged from the emergency room [24]. The observed association between public insurance and ICU utilization may be influenced by several factors, including differences in healthcare access, delayed care-seeking behaviors, and variations in the severity of illness at admission. Children with public insurance may face barriers to primary or specialty care, leading to more severe presentations upon hospitalization, or they may have underlying conditions that contribute to increased ICU needs. By examining this relationship, our study contributes to a broader understanding of how payer source may influence pediatric COVID-19 outcomes and hospital resource utilization.

However, some findings in our project were inconsistent with the existing literature, such as the relationship between comorbidities and disease severity. Similar to adults, other studies have indicated that comorbidities increase the likelihood of staying in the hospital longer [5,23,24]. A study by Antoon et al. (2021) found that obesity/type 2 diabetes mellitus; asthma; immunodeficiency; cardiovascular, pulmonary, neurologic/neuromuscular conditions; and Pulmonary Complex Chronic Conditions (CCCs) increase the risk of hospitalization in children [24]. In 2022, Woodruff et al. found that the risk of severe COVID-19 was higher among children with chronic lung disease, neurologic disorders, cardiovascular disease, prematurity, or airway abnormality [23]. However, in this project, having at least one comorbidity was not significantly associated with the length of stay or admission to intensive care. Our findings may not have reached the level of significance due to Type II error, given our relatively low sample of children with comorbidities. The most common comorbidities present in our sample of hospitalized children align with the literature as they are related to metabolic disorders and cardiovascular problems.

Another inconsistency in the literature was the relationship between age and the severity of outcomes. While our results did not indicate any difference in the length of stay or use of intensive care by age, the existing literature found that there was an increase in severity in children younger than two years [5,25] and children older than ten years old [4,5]. Di Fusco and colleagues (2022) found in their sample of children 0–11 years old that children 5–11 were more likely to use intensive care, have longer hospital stays, and have higher readmission rates [26].

Finally, the ZIP Code-level SVI was not significantly associated with either length of stay or ICU usage. This might be due to the small sample size or the collinearity between SVI and race/ethnicity. Although the SVI was not statistically significant in our multivariate models, the literature indicates that children of racial/ethnic minority groups are at greater risk for hospitalization and severe illness. Thus, in preparation for future public health emergencies, we must build upon existing models, such as the CDC-SVI, to better identify children at risk of severe disease or death. For instance, in a study conducted in Indonesia, childhood comorbidities were added to social vulnerability models to help identify risk factors for poor health outcomes [27]. Additionally, a weighted model should be developed to better understand how each CDC-SVI domain contributes to outcomes.

### Limitations

There are several limitations to this project. First, the project data sample only included patients with inpatient hospitalization, which limits the ability to compare children receiving inpatient vs. outpatient care. However, our findings add to the literature on exploring the risk factors of severe COVID-19-related outcomes among children who were admitted for hospitalization due to COVID-19 infection. Second, since the data included only hospitalized patients who resided in Clark County, Nevada, there was a relatively small number of observations for the pediatric population, resulting in relatively low statistical power. For example, the small number of cases with a comorbidity (n = 16) may increase the likelihood of Type II error, potentially leading to non-significant findings despite the presence of a true association. Future research with larger sample sizes could further validate our findings. Third, potentially important factors like hospital characteristics were not included in the dataset and, therefore, could not be included as covariates in the models. As such, our results should be interpreted as an association, not causality. Fourth, the Elixhauser Comorbidity Index was originally developed for adult populations and has not been specifically validated for pediatric populations. As a result, some relevant pediatric-specific conditions may not have been fully captured. Future studies may benefit from using pediatric-specific comorbidity measures to enhance the accuracy of risk adjustment. Lastly, because of the sample size limitations, all non-White racial groups were combined into a minority status category; thus, we cannot make specific distinctions within the diverse racial and ethnic groups combined into one category. Despite these limitations, this project offers evidence of some critical factors associated with the most severe COVID-19-related outcomes.

## 5. Conclusions

In conclusion, this community project provides important insights into factors associated with severe outcomes in children hospitalized with COVID-19 in Clark County, Nevada. Our findings highlight significant disparities, with minority children experiencing longer hospital stays and a higher likelihood of ICU utilization compared to non-Hispanic White children. Additionally, children covered by Medicaid or government insurance were at greater risk for ICU use, underscoring the role of socioeconomic determinants in health outcomes during the pandemic. These findings align with the existing literature on pediatric COVID-19 disparities but also reveal inconsistencies, such as the limited impact of comorbidities and age in our analysis, likely influenced by sample size constraints.

Several strategies are recommended to better prepare for future public health emergencies: (1) Enhance strategies to identify socially vulnerable communities: Although the SVI was not significantly associated with outcomes in our project, future research should refine vulnerability models to incorporate child-specific factors, such as immunization status, comorbidities, lab results, and treatments, to improve risk prediction. For example, adapting models like the CDC’s SVI to include child-related variables, as demonstrated in studies like Adwiluvito et al. (2024), may enhance our ability to identify at-risk children [27]. Furthermore, weighted models could help clarify the contributions of individual SVI components to health outcomes [28]. (2) Develop targeted and culturally appropriate public health interventions adapted to the local context: In the short term, supporting preventive care programs like immunization and nutrition initiatives can mitigate risks [29]. Long-term strategies should prioritize funding for interventions addressing chronic conditions and improving healthcare access. Community-engaged strategies can be instrumental in developing equitable and effective public health efforts and campaigns [30]. (3) Continue to support research: Further studies are essential to deepen our understanding of disease severity, therapeutic interventions, and care protocols for children with existing comorbidities [29]. Continued research will provide critical evidence to guide public health strategies and resource allocation.

Despite limitations, including a relatively small sample size and the focus on a single geographic area, this community public health practice project contributes to the growing body of literature on pediatric COVID-19. It provides a foundation for addressing health disparities in future public health crises. Implementing these recommendations can help reduce inequalities and improve health outcomes for children during pandemics and other emergencies.

## Figures and Tables

**Table 1:** Descriptive characteristics of patients.

	N	Percent
**Age group**		
Age 0–4	153	40.69
Age 5–12	105	27.93
Age 13–17	118	31.38
**Gender**		
Male	203	53.99
Female	173	46.01
**Race/ethnicity**		
White	142	37.77
Minority	234	62.23
**Having at least one comorbidity ^†^**		
Yes	16	4.26
No	360	95.74
**Payer type**		
Medicaid/CHAMPUS/CHAMPVA *	220	58.51
Commercial insurer/PPO/HMO *	137	36.44
Other (Charity/Self-pay/Miscellaneous)	19	5.05
**COVID-19 and other related diagnoses as a primary diagnosis**		
Yes	108	28.72
No	268	71.28
**Having COVID-19 symptoms or any related conditions**		
Yes	131	34.84
No	245	65.16
**Social Vulnerability Index (SVI)**		
Low or moderate vulnerability	122	32.45
High vulnerability	254	67.55

* Abbreviation: CHAMPUS = Civilian Health and Medical Program of the Uniformed Services; CHAMPVA = Civilian Health and Medical Program of the Department of Veterans Affairs; PPO = preferred provider organization; HMO = Health Maintenance Organization. **^†^** Out of 376 children with a non-missing length of stay variable, 16 were reported as having at least one comorbidity: specifically, 1 with diabetes without chronic complications, 1 with hypertension (uncomplicated), 8 with a chronic pulmonary disease, and 9 with obesity.

**Table 2:** Factors associated with the hospital length of stay and ICU utilization.

	Length of Stay (n = 376)			ICU Utilization (n = 235)		
	Coef.	95% CIs	*p*-Value	aOR	95% CIs	*p*-Value
**Age group**						
Age 0–4	−0.436	−2.196, 1.324	0.552	1.557	0.537, 4.518	0.415
Age 5–12	−0.277	−2.459, 1.905	0.757	1.857	0.556, 6.198	0.314
**Gender**						
Female	0.199	−0.609, 1.006	0.555	0.746	0.499, 1.115	0.153
**Race/ethnicity**						
Minority	1.091	0.419, 1.762	0.009	1.719	1.092, 2.706	0.019
**Having at least one comorbidity**	10.059	−3.614, 23.731	0.117	1.235	0.439, 3.471	0.689
**Payer type**						
Medicaid/CHAMPUS/CHAMPVA	−0.346	−1.682, 0.990	0.535	2.439	0.987, 6.030	0.054
Other (Charity/Self-pay/Miscellaneous)	−0.592	−3.382, 2.197	0.609	0.846	0.336, 2.127	0.722
**COVID-19-related primary diagnosis**	−2.239	−6.794, 2.316	0.262	1.092	0.374, 3.186	0.872
**Having COVID-19 symptoms**	3.548	0.185, 6.911	0.042	2.313	0.372, 14.385	0.368
**SVI**						
High vulnerability	−0.281	−1.129, 0.567	0.433	0.936	0.603, 1.453	0.769
(Pseudo) R^2^	0.2693			0.1283		

Note: Reference groups for age group, gender, race/ethnicity, payer type, SVI, and year-quarter fixed effects are 13–17 years old, male, non-Hispanic White, commercial insurer/PPO/HMO, and low/moderate vulnerability, respectively. Abbreviation: ICU = intensive care unit; Coef = coefficient; aOR = adjusted odds ratio; CHAMPUS = Civilian Health and Medical Program of the Uniformed Services; CHAMPVA = Civilian Health and Medical Program of the Department of Veterans Affairs; PPO = preferred provider organization; HMO = Health Maintenance Organization. For model’s goodness-of-fit, we included the R^2^ for the length of stay and pseudo-R^2^ for the ICU utilization.

## Data Availability

The datasets presented in this article cannot be shared due to privacy protections, HIPAA compliance, and existing data use agreements with the State of Nevada that prohibit data sharing.
